# Multi-tissue RNA-seq and transcriptome characterisation of the spiny dogfish shark (*Squalus acanthias*) provides a molecular tool for biological research and reveals new genes involved in osmoregulation

**DOI:** 10.1371/journal.pone.0182756

**Published:** 2017-08-23

**Authors:** Andres Chana-Munoz, Agnieszka Jendroszek, Malene Sønnichsen, Rune Kristiansen, Jan K. Jensen, Peter A. Andreasen, Christian Bendixen, Frank Panitz

**Affiliations:** 1 Department of Molecular Biology and Genetics, Aarhus University, Tjele, Denmark; 2 Department of Molecular Biology and Genetics, Aarhus University, Aarhus, Denmark; 3 Kattegatcentret, Grenaa, Denmark; Xiamen University, CHINA

## Abstract

The spiny dogfish shark (*Squalus acanthias*) is one of the most commonly used cartilaginous fishes in biological research, especially in the fields of nitrogen metabolism, ion transporters and osmoregulation. Nonetheless, transcriptomic data for this organism is scarce. In the present study, a multi-tissue RNA-seq experiment and *de novo* transcriptome assembly was performed in four different spiny dogfish tissues (brain, liver, kidney and ovary), providing an annotated sequence resource. The characterization of the transcriptome greatly increases the scarce sequence information for shark species. Reads were assembled with the Trinity *de novo* assembler both within each tissue and across all tissues combined resulting in 362,690 transcripts in the combined assembly which represent 289,515 Trinity genes. BUSCO analysis determined a level of 87% completeness for the combined transcriptome. In total, 123,110 proteins were predicted of which 78,679 and 83,164 had significant hits against the SwissProt and Uniref90 protein databases, respectively. Additionally, 61,215 proteins aligned to known protein domains, 7,208 carried a signal peptide and 15,971 possessed at least one transmembrane region. Based on the annotation, 81,582 transcripts were assigned to gene ontology terms and 42,078 belong to known clusters of orthologous groups (eggNOG). To demonstrate the value of our molecular resource, we show that the improved transcriptome data enhances the current possibilities of osmoregulation research in spiny dogfish by utilizing the novel gene and protein annotations to investigate a set of genes involved in urea synthesis and urea, ammonia and water transport, all of them crucial in osmoregulation. We describe the presence of different gene copies and isoforms of key enzymes involved in this process, including arginases and transporters of urea and ammonia, for which sequence information is currently absent in the databases for this model species. The transcriptome assemblies and the derived annotations generated in this study will support the ongoing research for this particular animal model and provides a new molecular tool to assist biological research in cartilaginous fishes.

## Background

Marine cartilaginous fishes such as *Squalus acanthias* (spiny dogfish) are ureotelic organisms which synthetize and excrete urea as the final product of the nitrogen metabolism. Besides, they are also ureosmotic organisms with urea being the main osmolite. Consequently, they retain large amounts of urea in their plasma in order to increase the osmolarity and maintain their body fluids isosmotic or slightly hyperosmotic with respect to sea water. To achieve such high concentration of urea in their body, efficient pathways of urea synthesis and retention are crucial, with liver, kidneys, intestine and gills being the primary tissues involved in this process [1–10] reviewed in [11, 12].

There are two main biosynthetic pathways of urea synthesis in cartilaginous fishes: the ornithine urea cycle (O-UC) and the purine degradation pathway, the first being the predominant pathway [1, 7, 13]. Cartilaginous fishes reabsorb most of the urea in the nephrons from the primary urine and transport it to the blood vessels [2, 4, 12] while also reducing the loss of ammonia in the gills and kidney in order to synthetize urea [12, 14, 15]. This mechanism is in part facilitated by their unique kidney morphology coupled with the presence of several metabolite transporters such as urea transporters (UTs), Rh-glycoproteins, aquaporins as well as the Na^+^, K^+^ and Cl^-^ reabsorption levels [2, 12, 14, 16, 17].

In addition to urea, other osmolites including Na^+^ and Cl^-^, are also important to maintain the osmolarity in cartilaginous fishes. Therefore, euryhaline cartilaginous fishes such as spiny dogfish are able to modify their internal urea, Na^+^ and Cl^-^ concentration as a response to changes in the salinity of the environment [5, 18, 19]. This process is highly regulated with the involvement of several tissues including the rectal gland, kidneys, liver, intestine and the gills, reviewed in [19]. The rectal gland in particular has been studied in great detail, due to its capacity of excreting large amounts of NaCl. Its simple tissue structure, the presence of several ion transporters and an early development of standardized experimental protocols have made rectal gland a model system for transport of chloride and ion exchange [20–26].

A significant part of the current knowledge about osmoregulation comes from studies in spiny dogfish, confirming this shark species as a valuable animal model for osmoregulation in cartilaginous fishes [1, 4, 10, 14, 17, 27]. Additionally, spiny dogfish has been used in other scientific fields for example in the study of choroid plexus transport [28–30] and drug discovery, where squalamine, a compound discovered in the liver of this shark species with antimicrobial activity [31, 32] has been more recently reported to have additional therapeutic activities [33–35]. In addition, to improve the research in this species, embryo-derived cell lines have been established a decade ago [36–38].

Although spiny dogfish is used as a model in a variety of biological research studies, only limited sequence resources providing transcriptome data are publicly available [36, 39, 40]. These previous experiments relied on Sanger sequencing technology and as a consequence, at the time of writing this manuscript the existing data comprises only 32,562 ESTs for *S*. *acanthias* on record at the National Center for Biotechnology Information (NCBI). Apart from rectal gland tissue [39], transcript information on other individual tissues is lacking for spiny dogfish. Genomic and transcriptomic resources for this animal group and particularly for sharks remain scarce even though there have been previous studies generating sequence data for cartilaginous fishes [39, 41–45]. To overcome these limitations, we performed extensive transcriptome sequencing of this shark using next generation sequencing (NGS) technology.

The aim of the present work was to establish a comprehensive characterization of the transcriptome of spiny dogfish based on high quality RNA-seq reads from kidney, liver, brain and ovary. One objective was to provide an extended molecular resource for the shark spiny dogfish while the other goal was to demonstrate how this novel data can be applied to investigate and improve insight into osmoregulation in this species. To ensure that both aspects, the more general one of generating a resource as well as the more focused one of creating data for specific interests like osmoregulation, were met we performed RNA-seq from kidney and liver with regard to the latter aspect while brain and ovary were chosen to provide a better description of the transcripts in this shark species. Following *de novo* assembly with Trinity [46, 47], the resulting assemblies were functionally annotated using the Trinotate pipeline (http://trinotate.github.io/)). Finally, the utility of the novel sequence and annotation resource to improve knowledge in spiny dogfish sharks was shown by investigation of the presence of genes involved in osmoregulation.

## Results

### RNA-seq and data processing

About 600 million raw reads were generated for spiny dogfish using the Illumina HiSeq2000 platform. Ribosomal and mitochondrial reads were removed and after trimming about 380 million high quality paired-end reads longer than 50bp remained; see Table 1 for detailed reports.

**Table 1:** Raw data processing.

Tissue	Raw	non-rRNA	non rRNA nor mitochondrial	Clean
Brain	103,564,548	103,374,482	72,383,520	63,079,760
Kidney	184,599,338	184,054,918	122,041,030	107,029,448
Liver	244,987,738	244,816,454	190,393,898	162,133,640
Ovary	63,688,000	63,387,924	57,334,040	50,820,452
Total	596,839,624	595,633,778	442,152,488	383,063,300

Total number of paired-end reads in the four RNA-seq experiments during the different filtering steps

### *De novo* assembly and assessment of the transcriptome assemblies

Using the Trinity assembler on the cleaned RNA-seq of the individual tissues, a total of 346,582 (brain); 316,366 (kidney); 226,007 (liver) and 291,104 (ovary) transcripts were generated. The combined assembly on the merged reads from all four tissues yielded in 705,797 transcripts. Aligning the read pairs back to the respective assemblies resulted in at least 70% of the reads mapping as proper pairs. In order to evaluate the completeness of the assemblies, the BUSCO pipeline was performed against a dataset of 3,023 conserved genes in vertebrates [48]. This analysis showed that the completeness varies between the assemblies reaching 87% completeness in the combined assembly. Finally, using blastx [49] the assemblies were aligned against the proteome of the holocephalan elephant shark (*Callorhinchus milii*), the only cartilaginous fish for which a genome assembly is available [41]. The number of significant unique hits (e-value cut-off 1e-20) in elephant shark that were covered by 70% of their length by a transcript from the different assemblies varied from 45% to 53%. A summary of the quality assessment of the assemblies is shown in Table 2.

**Table 2:** Assembly metrics and quality assessment of the transcriptome assemblies.

Feature	Brain	Kidney	Liver	Ovary	Combined	Previously known ESTs
Trinity transcripts	346,582	316,366	226,007	291,104	705,797	33,267 ^a^
Trinity genes	295,242	274,538	194,853	254,001	586,693	22,410 ^b^
N50	925 bp	778 bp	697 bp	699 bp	866 bp	651 bp
Average length	605 bp	570 bp	560 bp	555 bp	616 bp	601 bp
Mapped reads ^c^	78.79%	69.97%	76.08%	71.66%	71.77%	-
BUSCO ^d^	78% (10%)	76% (10%)	62% (10%)	67% (10%)	87% (7%)	18% (10%)
Elephant shark coverage ^e^	36,642 (52.6%)	36,132 (49.6%)	33,729 (44.9%)	35,691 (46.9%)	27,019 (59%)	8,301 (17.2%)

^a^ Total number of ESTs (recorded at NCBI for *S*. *acanthias*, plus sequences reported by [39]).

^b^ Clustering of (a) with cd-hit at 90% identity.

^c^ Percentage of reads properly mapped back to the transcriptome.

^d^ Proportion of complete and fragmented (in brackets) conserved vertebrate core genes according to the total number of core genes in the different datasets as derived by BUSCO analysis.

^e^ Number of unique hits in the elephant shark proteome; in brackets the percentage of best unique hits to elephant shark proteins covered by at least 70% in length by a transcript from the different assemblies or ESTs.

We filtered the transcripts by discarding potential contaminants, fragment sequences shorter than 300 bp and transcripts representing potential assembly artefacts (indicated by a FPKM value of zero) yielding a final set of 155,274 (brain), 140,336 (kidney), 110,527 (liver), 125,806 (ovary) and 362,690 (combined) transcripts, respectively. The stringent filtering increased the N50 values by about 50% while the completeness of the assemblies decreased only minimally as determined by BUSCO analysis using a set of conserved vertebrate genes. The number of filtered full-length cDNAs (transcripts classified by Transdecoder as ‘complete’) in the spiny dogfish transcriptomes amount to 24738 in brain (S1 File), 21129 in kidney (S2 File), 14650 in liver (S3 File) and 18109 in ovary (S4 File), respectively. An overview of the metrics of the different assemblies after filtering is shown in Table 3.

**Table 3:** Transcriptome assembly metrics after filtering.

	Brain	Kidney	Liver	Ovary	Combined
Trinity transcripts	155,274	140,336	110,527	125,806	362,690
Trinity genes	126,217	115,179	94,567	105,738	289,515
N50	1,586	1,439	1,196	1,337	1,302
Average length	950 bp	901 bp	823 bp	872 bp	866 bp
BUSCO	77%	74%	61%	67%	87%
Predicted proteins	69,346	62,185	46,198	51,013	123,110
Average length	310 aa	291 aa	270 aa	300 aa	287 aa

Assembly metrics for the assemblies after filtering.

### Annotation of the *de novo* assembled transcriptomes

**Fig 1:**
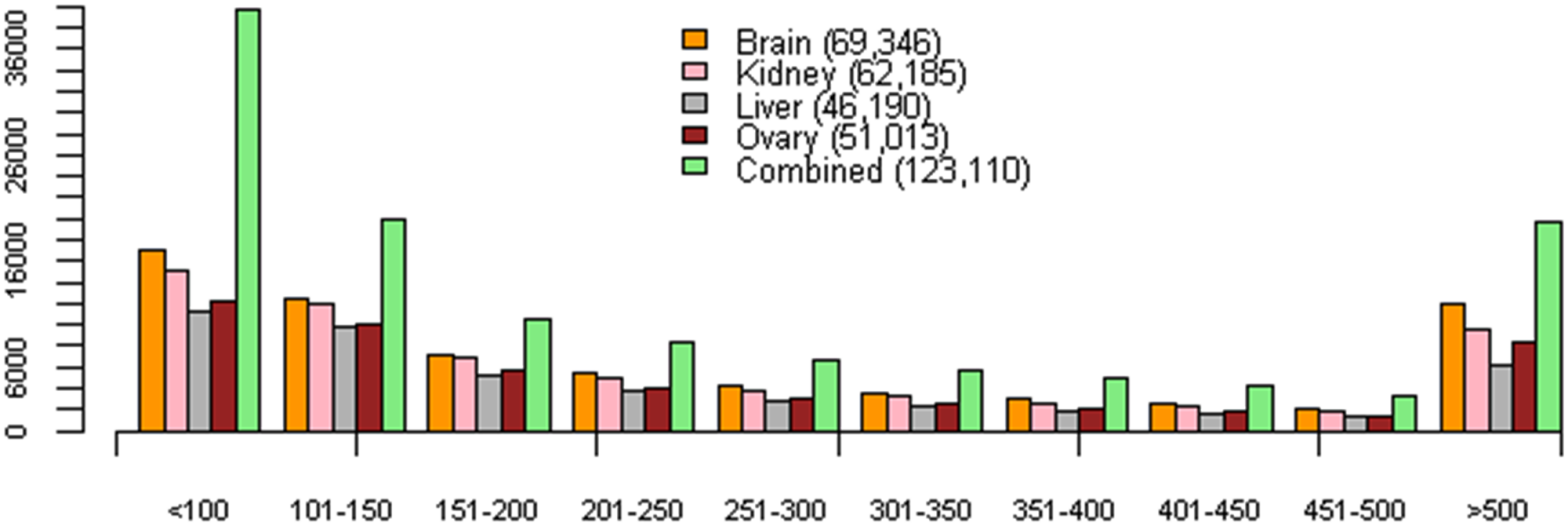
Length range and tissue distribution of the predicted proteins in the different assemblies. The total number of predicted proteins is shown in brackets. The x-axis represents the length range in amino acids while the y-axis corresponds to the total counts of the different predicted proteins.

Using TransDecoder (https://transdecoder.github.io/), a total of 69,346 (brain), 62,185 (kidney), 46,198 (liver), 51,013 (ovary) and 123,110 (combined) peptides longer than 60 amino acids were predicted (Fig 1 and Table 3). In order to estimate the overlap with known proteins, the predicted peptides were compared against the SwissProt, UniRef90 and Pfam databases. The percentage of predicted proteins with a blast hit in SwissProt was in the range of 71–72% in the assemblies of the individual tissues while this percentage in the combined assembly was 61%. Accordingly, the use of the larger protein database UniRef90 revealed a higher percentage of predicted proteins with a hit, ranging from at least 75% in the individual assemblies to 67% in the combined assembly. In addition, almost 50% of the predicted proteins had a hit in the Pfam database above the per domain noise score cutoff. SignalP predicted around 5% of the proteins having a signal peptide while TMHMM found that at least 11% of the proteins of each assembly carry at least one transmembrane region. Details of the annotation results are shown in Table 4. A graphical summary of the annotation results is shown in S1 Fig.

**Table 4:** Trinotate annotation of the transcriptome assemblies.

Anotation feature	Brain	Kidney	Liver	Ovary	Combined
Predicted proteins	69,346	62,185	46,198	51,013	123,110
SwissProt blastx	60,250	55,074	41,614	44,680	100,636
SwissProt blastp	49,522	44,782	33,288	36,813	78,679
Uniref90 blastx	67,066	61,204	46,371	49,763	115,108
Uniref90 blastp	52,177	47,380	35,088	38,809	83,164
Pfam	38,256	34,073	25,162	28,578	61,215
SignalP	3,700	2,979	2,212	2,808	7,208
TMHMM	8,793	7,443	5,227	6,189	15,971
eggNOG	24,954	23,905	18,635	19,429	42,078
GO	49,256	45,594	35,910	38,770	81,582

Number of transcripts and predicted proteins with a hit in the different databases searched: SwissProt, UniRef90 and Pfam. Signal peptide and transmembrane regions predictions were performed with SignalP and TMHMM software. GO and eggNog annotation were retrieved from blast and Pfam results.

### Identification of orthologues to elephant shark

**Fig 2:**
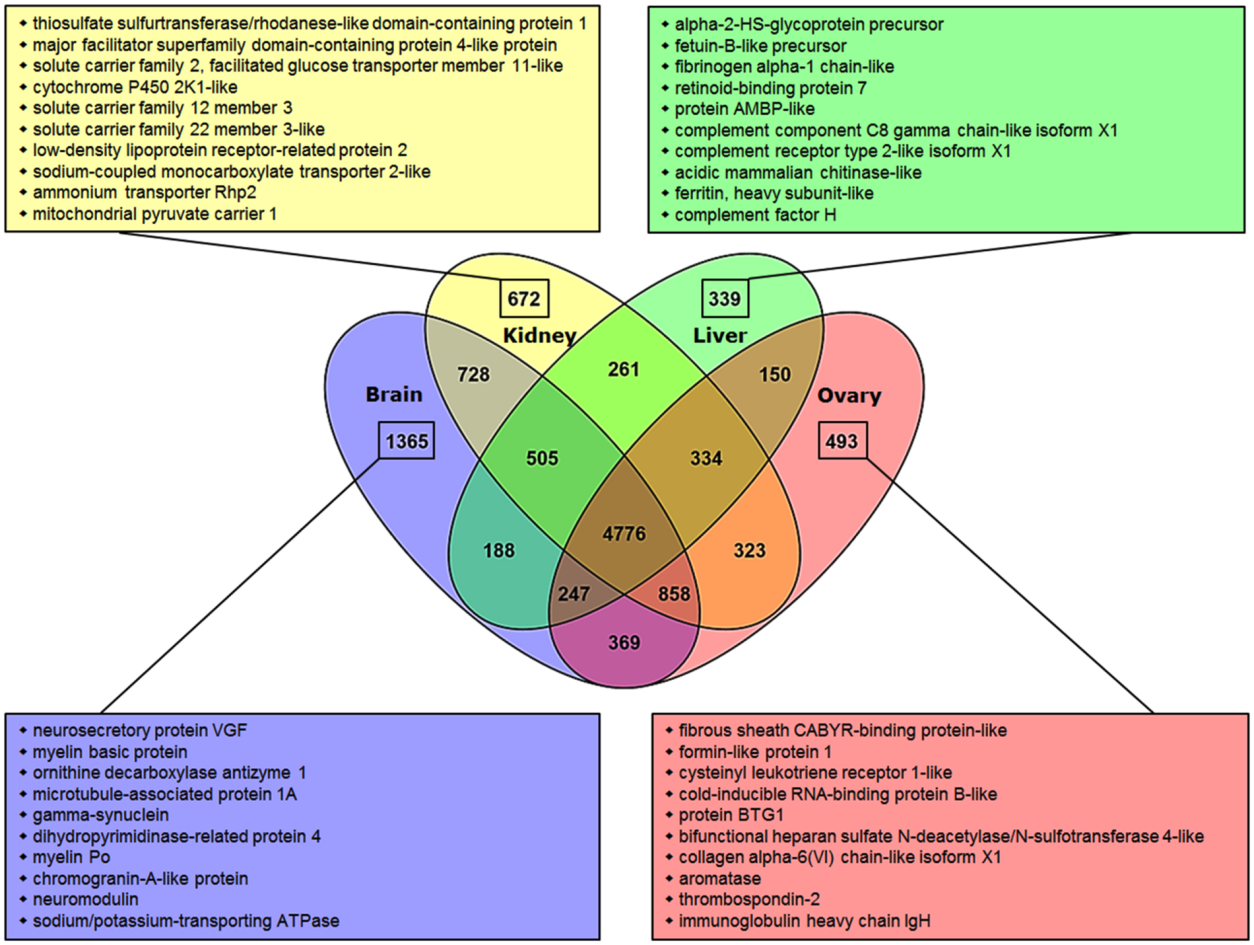
Relationship between elephant shark and spiny dogfish orthologs in the brain, kidney, liver and ovary transcriptome assemblies. Colored squares show the elephant shark annotation for the ten most expressed spiny dogfish one-to-one orthologs (measured in FPKM) unique to each assembly.

After protein prediction and redundancy removal (see Methods), the percentage of non-redundant elephant shark proteins with a homolog in the different spiny dogfish assemblies was 84% (brain), 82% (kidney), 72% (liver), 76% (ovary) and 90% (combined), respectively. From those, 9,036 (brain), 8,457 (kidney), 6,800 (liver), 7,550 (ovary) and 10,623 (combined assembly) corresponded to one-to-one orthologous pairs. (Fig 2). As expected, the encoded proteins unique to the respective assembly are related to their specific tissue function in sharks: For instance, neurosecretory proteins and myelin basic protein in brain, several members of the cystatin family protein, ferritin and several zymogens in liver and different solute carriers in kidney. In ovary, apart from genes associated with reproduction (such as fibrous sheet CABYR-binding protein like and aromatase among others) we note the presence of genes involved in the immune system such as cysteinyl leukotriene receptor 1-like, protein BTG1 and immunoglobulin heavy chain IgH, which is explained by the specific shark anatomy where the ovaries are enclosed in the epigonal organ, a part of the lymphomyeloid system in sharks [50, 51].

### Spiny dogfish shark annotated transcriptome assemblies: A molecular tool

In order to assess the usability of our transcriptome assemblies as a molecular tool, we investigated the presence of genes involved in osmoregulation, a field where the spiny dogfish is of great interest as a model system. To this end the Trinotate annotation reports (S1–S5 Tables) were searched for genes, which are involved in urea synthesis as well as urea and ammonia transport as the main processes participating in osmoregulation in cartilaginous fishes.

#### Urea synthesis

To identify transcripts, which were assigned to the known gene ontology terms related to urea synthesis biosynthetic pathways, we queried the Trinotate annotation reports for the gene ontology terms “urea cycle” (GO:0000050), “allantoin catabolic process” (GO:0000256) and “urate oxidase activity” (GO:0004846). Since the transporter CPSIII in cartilaginous fishes uses glutamine instead of ammonia as substrate [3, 52], the term “glutamate-ammonia ligase activity” (GO:0004356) was also inquired. The best scoring hits to SwissProt and Uniref90 databases for these transcripts showed proteins involved in urea biosynthetic pathways. To enable comprehensive gene identification, we also compared the transcripts found in the different assemblies with the elephant shark, the closest organism with an available genome assembly [41]. To this end, the proteome of the elephant shark was used as reference and elephant shark—spiny dogfish orthologous groups were identified. We found all the genes involved in purine degradation pathways and ornithine-urea cycle to be present in the liver assembly with the exception of one arginase gene (Arg 1-like). However, some other tissues such as kidney present many of the genes involved in these urea biosynthetic pathways. The results are summarized in Table 5. Detailed annotation for the genes and isoforms involved in urea biosynthesis that were identified from the annotation reports is provided in S6 Table, while the elephant shark—spiny dogfish orthologous groups of urea synthesis genes are listed in S8 Table; spiny dogfish predicted protein sequences involved in the urea cycle are given in S5 File. Importantly, blast searches against NCBI *Squalus acanthias* nucleotide, ESTs and protein databases revealed that in the case of Uox, Ornt1, Gs2, Aln, Allc, and Arg2 the transcripts identified code for proteins for which our sequences represent novel information which has not been described before. Moreover, with the exception of CPSIII, the remaining spiny dogfish transcripts identified for these biosynthetic pathways either code for a longer version of the protein or are not fully covered by the existing spiny dogfish sequences indicating that our RNA-seq and transcriptome resources provide additional novel sequence information for these genes (see S9 Table).

**Table 5:** Genes involved in osmoregulation identified in the different spiny dogfish transcriptome assemblies based on orthologous proteins between elephant shark NCBI proteins and spiny dogfish assemblies.

	Gene products	Brain	Kidney	Liver	Ovary
Purine degradation pathway	Aln	-	-	+	-
	Allc	-	+	+	-
	Uox	-	-	+	-
O-UC	Arg-1-like	-	+	-	-
	Arg-2	*	+	+	+
	Otc	*	+	+	+
	Ass1	+	+	+	+
	Asl	+	+	+	+
	CpsIII	-	-	+	-
	Gs1	+	+	+	+
	Gs2	-	+	+	+
Accessory O-UC	Ornt1	+	-	+	+
	Nags	-	-	+	-
Urea transport	Ut-1	*	+	-	-
	Aqp3-like	-	+	-	-
	Aqp3-like	-	+	-	-
Water transport	Aqp1-like	+	-	-	-
	Aqp9	-	+	+	-
	Aqp4	-	+	-	-
	Aqp15	-	+	-	-
Rh-glycoproteins	Rhag	+	+	+	+
	Rhbg	+	+	+	-
	Rhcg ^a^	*	*	*	-
	Rhp2	-	+	*	-
	Rh30-like	*	-	-	-

Aln (allantoinase), Allc (allantoicase), Uox (urate oxidase), Arg (arginase), Otc (ornithine transcarbamylase), Ass (argininosuccinate synthase), Asl (argininosuccinate lyase), CpsIII (carbamoyl phosphate synthetase III) and Gs (glutamine synthetase), Ornt1 (ornithine-citrulline mitochondrial transporter) and Nags (N-acetyl synthase) which likely acts as cofactor in CPSIII in cartilaginous fishes [11, 52], Ut-1 (urea transporter) and Aqp (aquaporins).

^a^ Possible orthologue to elephant shark. (+) Spiny dogfish predicted proteins covering >70% or <70% (*) of the length of the hit to the elephant shark orthologue. (-) No gene product identified. Accession numbers and transcript IDs for elephant shark proteins and the predicted proteins of the *de novo* assemblies are found in S8 Table.

#### Urea transport

**Fig 3:**
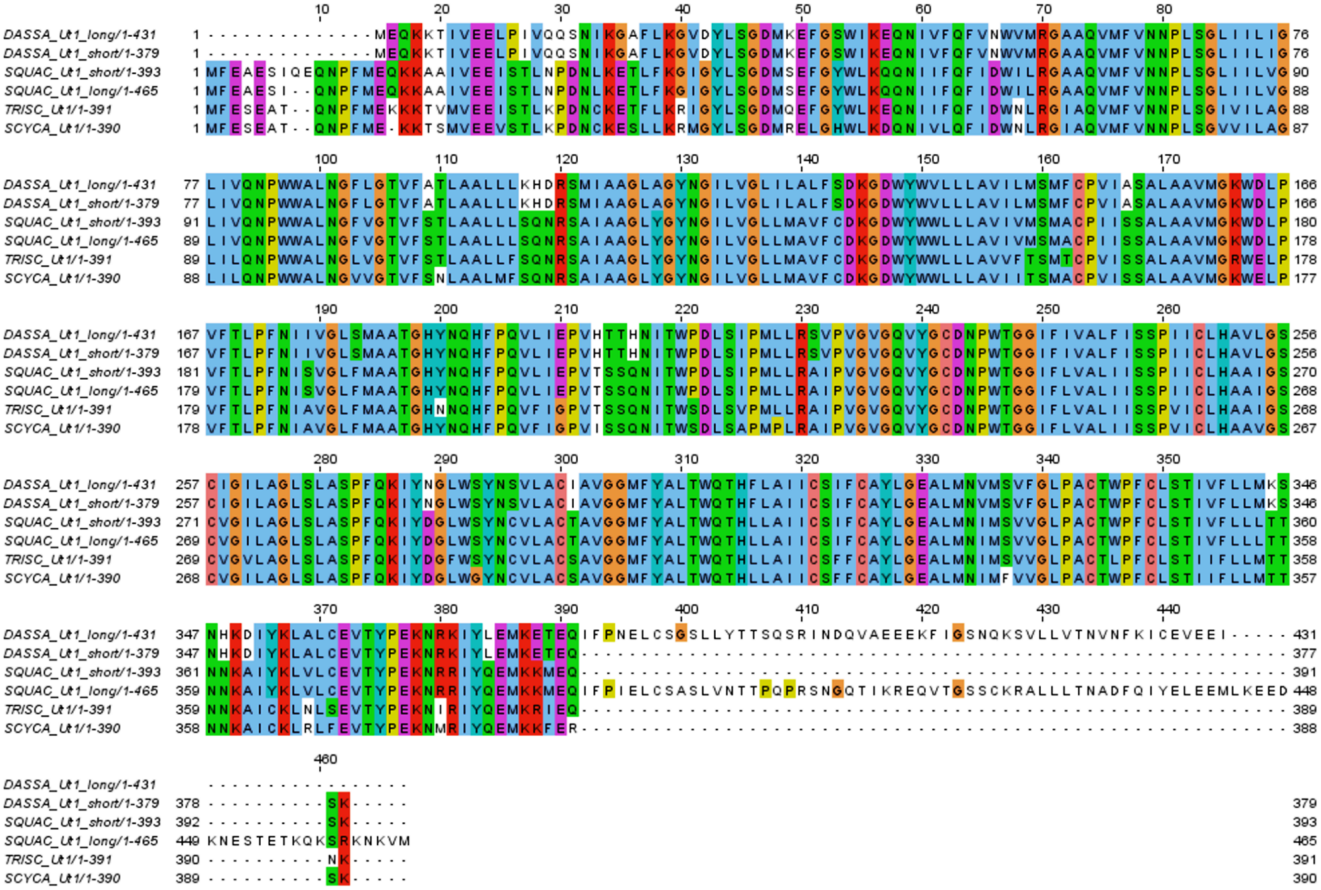
Multiple alignments of Ut1 proteins in selected elasmobranchs. Atlantic stingray (*Dasyatis sabina*; DASSA), spiny dogfish (*Squalus acanthias*; SQUAC) kidney isoforms, banded houndshark (*Triakis scyllium*; TRYSC) and lesser spotted catshark (*Scyliorhinus canicula*; SCYCA). Alignment performed with MUSCLE showing ClustalX color scheme at 30% conservation threshold. Accesion numbers for DASSA_Ut1_long (AAM46683.1), DASSA_Ut1_short (AAQ07592.1), TRISC_Ut1 (BAC75980.1) and SCYCA_Ut1 (AEH59797.1). Protein IDs for Ut1_long (TR103653|c5_g2_i2|m.138919), Ut1_short (TR103653|c5_g2_i1|m.138917) identified in the *de novo* assemblies.

**Fig 4:**
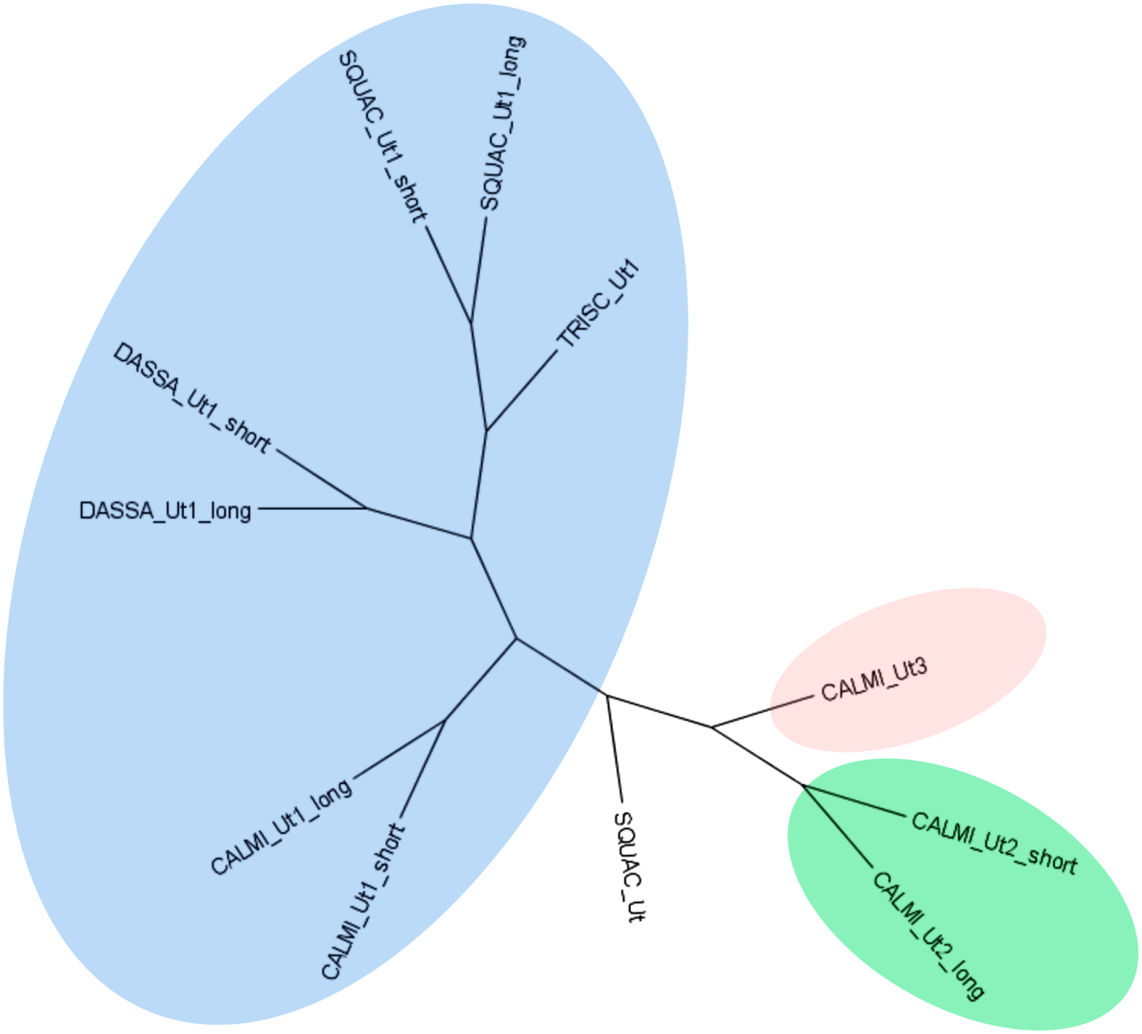
Phylogenetic tree of multiple urea transporter proteins in selected elasmobranchs and elephant shark. Ut1 (blue), Ut2 (green) and Ut3 (pink). *Dasyatis sabina* (DASSA), *Squalus acanthias* (SQUAC), *Triakis scyllium* (TRYSC), *Scyliorhinus canicula* (SCYCA) and *Callorhinchus milii* (CALMI). SQUAC_Ut1_long and SQUAC_Ut1_short correspond to the Ut isoforms identified in the kidney assembly (TR103653|c5_g2_i2|m.138919 and TR103653|c5_g2_i1|m.138917, respectively). SQUAC_Ut corresponds to the isoform identified in brain (TR95738|c0_g3_i3|m.93774). Accesion numbers for DASSA_Ut1_long (AAM46683.1), DASSA_Ut1_short (AAQ07592.1), TRISC_Ut1 (BAC75980.1), SCYC_ Ut1 (AEH59797.1), CALM_ Ut 1_long (BAH58773.1), CALMI_Ut 1_short (BAH58774.1), CALMI Ut 2 long (BAH58775.1), CALMI Ut 2 long (BAH58776.1) and CALMI Ut 3 (BAH58777.1).

Annotation reports were searched under the gene ontology terms “urea transmembrane transporter activity” (GO:0015204), “urea transport” (GO:0015840) and “urea transmembrane transport” (GO:0071918). The resulting transcripts code for proteins that are orthologues to the elephant shark urea transporter efUT1 as well as to two different aquaporin genes (Aqp3-like) proteins. In the case of the urea transporter, two isoforms of *ut1* were identified in the kidney assembly: the first one encodes an identical protein sequence to the previously described urea transporter in spiny dogfish shUT [53] while the second one codes for a longer protein and possesses an extended carboxyl terminal part similar to the one identified in the Atlantic stingray *Dasyatis sabina* [54, 55], *s*ee Fig 3. In addition, three more urea transporter transcripts were found in the spiny dogfish brain transcriptome belonging to the same gene assembled by Trinity. However, the transcript coding for the longest peptide with a partial length encoding 272 amino acids (TR95738|c0_g3_i3|m.93774; see S6 File) is also an orthologue of efUT1, lacks strong similarity to the Ut1 isoforms identified in the spiny dogfish kidney assembly and does not cluster in the same group as the other Ut1 proteins (Fig 4), making it difficult to assess whether it is an Ut1 isoform or a protein encoded by a novel gene. Finally, the two transcripts coding for Aqp3-like proteins were only found in the spiny dogfish kidney assembly (Table 5). Blast searches confirmed that their best hit in the existing NCBI *S*. *acanthias* databases is Aqp4 but with only 27% sequence identity, thus indicating that of these two genes identified have not previously been described for spiny dogfish.

To further investigate the presence of additional aquaporin genes, the transcriptome assemblies were searched for transcripts coding for the aquaporin family protein domain “major intrinsic protein” (PF00230.15). We observed only one Trinity gene coding for that protein domain in each of the brain, liver and ovary transcriptomes while we found 9 different in kidney (S7 Table). Elephant shark—spiny dogfish orthologous pairs revealed that these transcripts code for proteins that are orthologues to elephant shark Aqp1-like, Aqp9, Aqp4, Aqp-fa-chip, Aqp0 (lens fiber major intrinsic protein-like) and to the two different *A*qp3-like proteins above described (S8 Table; spiny dogfish predicted protein sequences for aquaporins are provided in S7 File). An *aqp1-like* transcript was only found in brain, while *aqp9* transcripts were identified in kidney and liver. On the other hand, the remaining aquaporin transcripts were only identified in the kidney assembly (Table 5). Blast searches against nucleotide and protein databases for *S*. *acanthias* showed that the partial transcript, which is orthologous to elephant shark aquaporin-FA-CHIP encoded a protein identical to Aqp15, previously described in spiny dogfish [56]. Even though the identified Aqp1-like, Aqp4, Aqp9 and Apq15 align to the known ESTs, nucleotides or proteins for spiny dogfish, the resulting predicted proteins improve the existing spiny dogfish sequences and refine information in the NCBI databases (see S9 Table). Taken together, completely novel sequences are presented for Aqp3-like and Aqp0 while the sequence information for Aqp1-like and Aqp9 is also improved based on the *de novo* assemblies. In the case of Aqp15 and Aqp4 [57], however, the predicted proteins were identical to the ones already described.

Finally, the presence of several sodium, potassium and chloride transporters was assessed in the kidney assembly by means of transcripts assigned to the gene ontology terms listed in Table 6 and S7 Table. The elephant shark—spiny dogfish orthologue relationship revealed 82 orthologues using these gene ontology terms in the kidney assembly (data not shown). However, the functional roles of these genes in shark kidney will have to be investigated in further studies.

**Table 6:** Gene ontology (GO) terms searched in the kidney assembly related with Na^+^, K^+^ and Cl^-^ transport.

GO term	Description
GO:0008511	Sodium: potassium: chloride symporter activity
GO:0006813	Potassium ion transport
GO: 0070294	Renal sodium ion absorption
GO:0035725	Sodium ion transmembrane transport
GO:1902476	Chloride transmembrane transport
GO:0006821	Chloride transport
GO:0006814	Sodium ion transport
GO:0002028	Regulation of sodium ion transport
GO:0003096	Renal sodium ion transport

#### Ammonia transport

**Fig 5:**
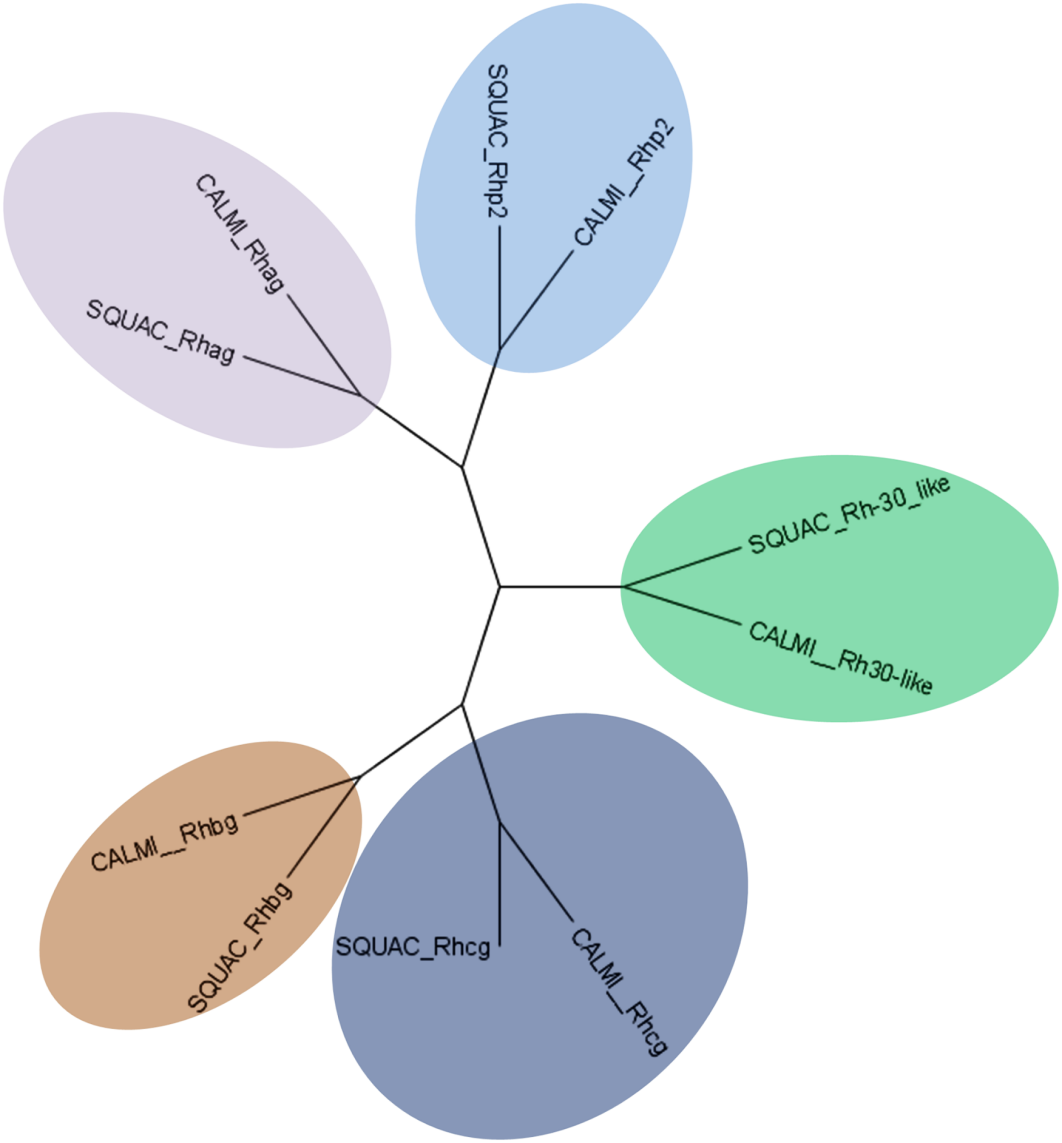
Phylogenetic tree of the different Rh-glycoproteins found with their elephant shark orthologues. Accession numbers: CALMI_Rhag (AFO95352.1), CALMI_Rhbg (AFP03342.1), CALMI_Rhcg (XP_007906553.1), CALMI_Rhp2 (AFP00304.1), CALMI_Rh30-like (NP_001279904.1). Protein IDs: SQUAC_Rhag (TR44094|c0_g1_i1|m.17165), SQUAC_Rhbg (TR9088|c0 g1 i1|m.3455), SQUAC_Rhcg (TR88392|c0_g1_i1|m.62153), SQUAC_Rhp2 (TR33065_c0_g1_i1_m.11704), SQUAC_Rh30-like (TR93310|c0 g1 i2|m.80440). Colors showing groups of Rhag (grey), Rhbg (brown), Rhcg (dark blue), Rhp2 (light blue) and Rh-30-like (green).

Transcriptome annotation revealed several genes coding for ammonium transporter protein domains in brain (5), kidney (4), liver (6) and ovary (2), which align to known Rh-glycoproteins such as Rhag, Rhbg, Rhcg and Rhp2 in SwissProt and UniRef 90 databases (S7 Table). Orthologous pairs between elephant shark and our *de novo* assembled spiny dogfish transcriptomes confirmed the identity of those Rh-glycoproteins in spiny dogfish (see Table 5 and S8 Table). From those, Rhag, Rhbg and Rhp2 exhibit 100% identity to the same proteins previously identified in spiny dogfish [14]. Moreover, transcripts coding for orthologues to elephant shark Rh30-like protein were found. The longest isoform of these transcripts is found in the brain assembly, which codes for an open reading frame (ORF) of 304 amino acids length (S8 File) with 64% identity to the elephant shark Rh30-like protein. Additionally, we found a partial transcript in the brain assembly encoding an open of 337 amino acids (TR88392|c0_g1_i1|m.62153; see S8 File) whose best Uniref90 hit is the elephant shark protein Rhcg. Although this protein was neither included in any orthologue group between elephant shark and spiny dogfish, it shares 72% identity with the same elephant shark protein. Altogether, our transcriptome resources provided evidence for the putative Rhcg sequence being completely new for spiny dogfish in addition to longer predicted peptide sequences, e.g. the predicted Rhag is 83 amino acids longer in the carboxyl-terminal part and the predicted Rh30-like is 101 amino acids longer in the amino-terminal part. The predicted proteins identified as orthologues to elephant shark Rhbg and Rhp2, however, are fully covered in the existing *S*. *acanthias* databases. The phylogenetic tree of the Rh-glycoproteins identified (Fig 5) shows, as expected, that all of the identified Rh-glycoproteins cluster with their respective orthologs in the elephant shark.

## Discussion

The high number of the spiny dogfish predicted proteins align to the different databases searched provide a more complete annotation than obtained by previous studies involving other shark transcriptomes [43–45]. Although the activities of all the enzymes involved in urea synthesis in spiny dogfish have been biochemically studied since the 1960s [1], sequence information of the genes that code for those proteins is still incomplete in this shark species. The analysis of the spiny dogfish transcriptome reveals the presence of all the genes involved in the two main biosynthetic pathways for urea synthesis in cartilaginous fishes (Table 5) and as expected, all of these genes are expressed in the liver, the main tissue involved in urea synthesis. However, in accordance with previous studies [6–8, 58] some of the urea synthesis genes were identified in extra-hepatic tissues as well.

**Fig 6:**
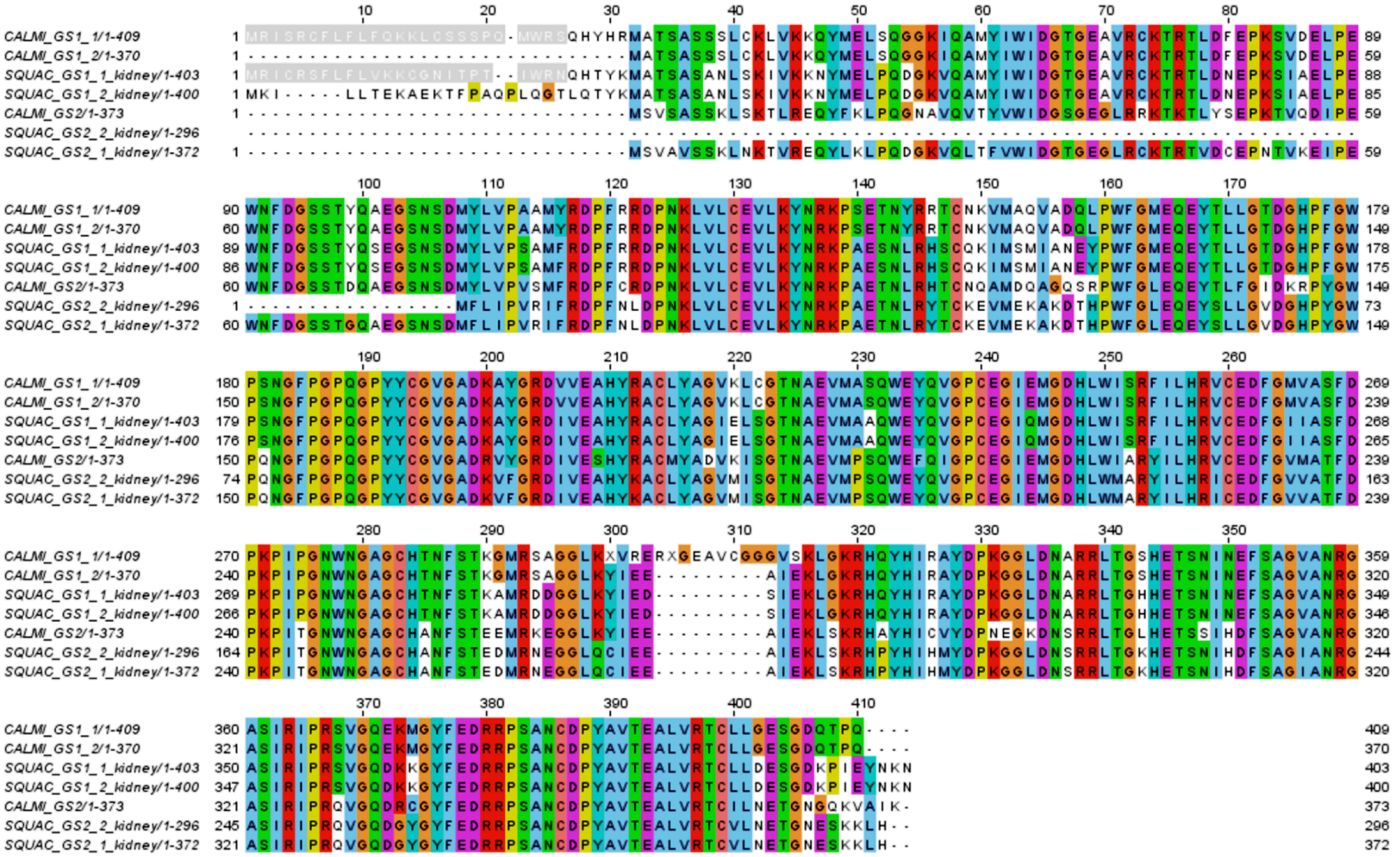
Multiple alignments of the different glutamine synthetase (Gs) in elephant shark (CALMI) and spiny dogfish (SQUAC). Mitochondrial localization signals are highlighted in grey. Alignment performed with MUSCLE showing ClustalX color scheme at 30% conservation threshold. Protein IDs and accession numbers for SQUAC_GS1_1_kidney (TR71041|c0_g1_i1|m.31201), SQUAC_GS1_2_kidney (TR71041|c0_g1_i2|m.31207), SQUAC_GS2_1_kidney (TR91001|c0_g1_i3|m.57038), SQUAC_GS2_2_kidney (TR91001|c0_g1_i1|m.57036), CALMI_GS1_1 (XP_007901126.1) and CALMI_GS1_2 (BAK96221.1), CALMI_GS2 (BAK96222.1).

Earlier studies have described one glutamine synthetase (*Gs*) in spiny dogfish [59], being orthologous to elephant shark Gs1. In the present work we were able to identify two different glutamine synthetases in the spiny dogfish that are orthologues to the two glutamine synthetases previously identified in the elephant shark [8] termed Gs1 and Gs2 (Fig 6).

Comparison with public resources for spiny dogfish failed to retrieve a sequence similar to the *gs2* transcripts identified in the present work, therefore, to our knowledge this is the first study in which a second *gs* gene in this species was predicted. In accordance with the findings in elephant shark [8] and the existing sequence information for *S*. *acanthias*, we predict several *gs*1 isoforms coding for alternative N-terminal parts carrying and lacking mitochondrial localization signals. On the other hand, unlike the in the elephant shark we do find several *gs2* isoforms in spiny dogfish for which in all cases the predicted protein lacks the mitochondrial sequence signal. Previous studies have described that the expression of these multiple genes varies among tissues and developmental stages in the elephant shark [8], suggesting differentiation of the role of the different isoforms. The biological implication of these findings in spiny dogfish and in elasmobranchs needs further investigation.

Regarding the transcripts coding for arginase proteins, we were able to identify transcripts coding for different arginases, Arg1 and Arg2 as described in the elephant shark [8]. Transcripts coding for Arg2 are found in the four tissues and their predicted proteins have not been described before in the spiny dogfish while the ones coding for Arg1-like are only found in the spiny dogfish kidney assembly and their predicted protein is identical to the previously identified arginase. The predicted Arg2 protein seems to be the predominant arginase in the O-UC although both arginases carry mitochondrial localization signals which is in accordance with the elasmobranch pathway of O-UC where the last step of urea synthesis occurs in the mitochondria [8, 10, 11].

With reference to urea transport and reabsorption genes, three different urea transporters (UT) have been described in the elephant shark, termed efUT1, efUT2, and efUT3 [60]. In comparison, only one gene has been reported for the spiny dogfish (shUT) which is the ortholog of efUT1 [53]. In the case of the Atlantic stingray the efUT1 ortholog codes for two isoforms, with the second one showing an extended carboxyl terminal part [54, 55]. Analogously to the Atlantic stingray, we identified a novel and longer isoform of shUT in spiny dogfish which has been suggested to play a role in the acclimation to different salinities for euryhaline elasmobranchs. Since spiny dogfish has been described euryhaline future studies are needed to investigate whether this second isoform participates in such acclimation.

Water retention has been suggested as an important step in urea reabsorption process in which aquaporins (AQP) play an active role [12]. Although the precise role of aquaporins in the transport of urea and ammonia is not well understood, human AQP3, AQP7 and AQP9 are known to be permeable to both ammonia and urea while AQP8 and AQP10 only to ammonia [61, 62]. We identified transcripts coding for Aqp1, Aqp4, Aqp9, and Aqp3 (two copies), all of which were found in the spiny dogfish kidney. The presence of two different Aqp3 genes in the kidney assembly is noteworthy; however, to determine the potential roles of these aquaporins in urea and ammonia reabsorption in cartilaginous fishes does require further investigation.

Apart from minimizing urea loss, sharks are able to diminish leakage of ammonia through the gills and the kidney [12, 14, 15]. Rh-glycoproteins are a group of proteins belonging to the ammonium transporter superfamily being able to transport ammonia [63]. They are believed to play a crucial role in the urea metabolism of cartilaginous fishes [12, 14, 15]. This protein family has five different members in elephant shark: Rhag, Rhbg, Rhcg, Rhp2 and Rh-30-like, of which only three, Rhag, Rhbg and Rhp2, have been described in spiny dogfish so far [14]. Our transcriptome analysis predicted proteins with complete sequence identity to these three proteins. In addition, we found partial length contigs coding for Rh-30 and a transcript that encoded a putative Rhcg, which sequence has not been previously described in spiny dogfish.

## Conclusions

This is the first study to generate large-scale NGS data and single tissue transcriptome characterization for the model species *Squalus acanthias*. We present an extensive multi-tissue transcriptome analysis and the annotation of our assemblies showed that the predicted proteins have a high percentage of significant hits in the SwissProt and UniRef90 databases. The completeness of the assemblies using BUSCO revealed that although we only sequenced four tissues, the combined assembly has a completeness of 87%. Querying annotation reports provided important new sequence information in genes involved in urea-based osmoregulation, a scientific field where elasmobranchs and particularly spiny dogfish as an animal model are of great interest.

Taken together, our data collected provides a new resource as well as an extended catalogue of genes involved in osmoregulation in spiny dogfish. Due to the detailed annotation generated, the same approach would be suitable to extract sequence information related to other areas of biological interests. Consequently, we believe the data generated will be of great support for the ongoing research in elasmobranchs and in spiny dogfish shark, particularly.

## Materials and methods

### Tissue sampling and ethics statement

The shark tissue samples were opportunistically obtained from the public aquarium Kattegatcentret, Grenå, Denmark independent of our study. Due to veterinary reasons the specimen was to be euthanized and we were contacted to gain access to the tissue material as a collaborative effort by the Kattegatcenter; following dissection samples were snap-frozen in liquid nitrogen and stored at -80°C. It is important to emphasize that the shark did not get euthanized as part of this investigation and that the tissue material became opportunistically available for research. Thus, no ethical approval or permit for animal experimentation was required, as the individual was not sacrificed specifically for this study.

### RNA-seq: Library preparation and sequencing

Total RNA was isolated from brain, kidney, liver and ovary applying the procedure for isolation of total RNA as provided by the mirVana miRNA Isolation Kit (Ambion) according to the manufacturer’s instructions. RNA was treated with Ribo-Zero rRNA removal kit (Epicentre) in order to remove rRNA from the RNA population. Libraries were prepared with a selected insert size of 300 bp using ScriptSeq v2 RNA-Seq Library Preparation Kit (Epicentre) for strand-specific and multiplexed libraries following manufacturer instructions. Paired-end sequencing was performed on the Illumina HiSeq2000 platform to a read length of 150 bp. De-multiplexing was performed with Illumina software allowing zero mismatches in the barcode. RNA-seq raw reads after de-multiplexing and removal of technical sequences by Illumina software have been deposited in the European Nucleotide Archive (ENA) database under the study accession PRJEB14721.

### Filtering of raw data

FastQC (http://www.bioinformatics.bbsrc.ac.uk/projects/fastqc) was used in order to get a visual overview of the read quality. Before assembly, possible remaining rRNA as well as mitochondrial reads were removed from the raw data based on hits against the LSU_Ref and SSU_Ref Silva databases version 119 [64] and the *Squalus acanthias* mitochondrial genome (accession NC_002012): Raw reads were mapped to both databases using Bowtie2 with the wrapper script provided by FastQScreen version v0.4.4 with default parameters (http://www.bioinformatics.babraham.ac.uk/projects/fastq_screen/). The reads that did not map to these indexes were considered non-ribosomal and non-mitochondrial and were used for downstream filtering steps.

Trimmomatic version 0.32 [65] was used to remove adapter sequences and trim low quality bases using the parameters–phred33 ILLUMINACLIP: Scriptseqv2_adapters:2:30:10 LEADING:3 TRAILING:3 SLIDINGWINDOW:4:15 MINLEN:50.

### *De novo* assembly

High quality paired-end reads were assembled using Trinity software version 2.0.6 with default parameters except–max_memory 15G –CPU 20 –SS_lib_type FR–min_kmer_cov 2 –KMER_SIZE 25 –min_contig_length 200 for the single tissue assemblies. For the combined assembly, reads from the four tissues were merged into a single fastq file and assembled together. The parameters applied were the same as for the individual assemblies except for the added option–normalize_reads. In both cases, the assembly metrics were obtained using the *Trinity_stats*.*pl* script from the Trinity software package.

### Back mapping of reads and abundance estimation

Reads were mapped back to the transcripts using the script *bowtie_PE_separate_then_join*.*pl* from the Trinity software 2.1.1; Bowtie [66] was run with the parameters–p 20 –all—best—strata—m 300. The percentage of reads that map to the assembly as properly paired, improper pairs, left only and right only was calculated with the script *SAM_nameSorted_to_uniq_count_stats*.*pl*. Abundance of each transcript and gene was calculated using the *align_and_estimate_abundance*.*pl* script from Trinity software 2.0.6. Default settings were used except for the options–est_method RSEM–aln_method bowtie–trinity_mode—prep_reference—SS_lib_type FR. RSEM version 1.2.19 [67] was used to estimate the abundance of each transcript.

### Assessment of full-length coverage of transcripts and completeness of the assemblies

Assemblies were aligned with blastx version ncbi-blast-2.2.30+ [49] to the elephant shark proteome (downloaded from NCBI protein database, as of January 28, 2016). Blastx output was filtered to an e-value of at least 1e-20. The percentage of the assembly query that covers a database hit was calculated using the Perl script bound to Trinity suite *analyze_blastPlus_topHit_coverage*.*pl*.

To check the completeness of the assemblies BUSCO software version 1.1 [48] with–trans option against the vertebrata dataset was used. BUSCO software and the vertebrata dataset were downloaded from http://busco.ezlab.org/. To run BUSCO, the programs ncbi-blast-2.2.30+ [49], HMMER version 3.1 [68] and EMBOSS 6.3.1 [69] were used.

### Filtering of assembled transcripts

Transcripts with zero fragments per kilo base per million reads (FPKM), transcripts per million (TPM), and IsoPct (percentage of the reads that align to each isoform over the reads that aligned to all the gene isoforms) were removed. A second filter was performed to remove transcripts shorter than 300 bp. Tabulated expression values for the genes of each tissue are available to allow more stringent filtering (S9, S10, S11 and S12 Files). In addition, the assemblies were screened for possible vector contamination by aligning them to the Emvec databank ftp://ftp.ebi.ac.uk/pub/databases/emvec/ using blastn with a cutoff of 90% identity and an e-value of 1e-10.

### Functional annotation of the transcriptome

Protein-coding transcripts were predicted with TransDecoder_r20140704 (https://transdecoder.github.io/) with–S–m 60 options and the rest parameters set as default. Trinotate version 2.0.0 was used to retrieve the functional annotation of the *de novo* assembled transcriptomes of spiny dogfish. In order to annotate the transcriptome, SwissProt, Uniref 90, Pfam databases and the Trinotate boilerplate compatible with those databases and Trinotate v2.0.0 were downloaded from the Trinotate webpage http://trinotate.github.io/ on October 23, 2015. Additionally, ncbi-blast-2.2.30+ [49] TMHMM software version 2.0 [70], HMMER version 3.1 [68] and SignalP version 4.1 [71] were used with the parameters recommended for Trinotate annotation. Finally, the annotation reports for the assemblies were generated using an e-value cutoff of 1e-05 and the per domain noise score in Pfam as cutoff. Gene Ontology (GO) [72] and eggNOG [73] annotations were retrieved from the blast results. In the case of GO, additional GO annotation was retrieved from the Pfam database results.

### Elephant shark orthologue assignment

The elephant shark proteome (downloaded from NCBI protein database, as of January 28, 2016) and the predicted proteins from the *de novo* assemblies were used for orthologue assignment after removing redundancy at 90% identity with cd-hit [74, 75]. In order to get a reliable set of orthologues between both datasets, only proteins with at least 100 amino acids of length where kept. Finally, Inparanoid version 4.1 [76] was used with default parameters and the options bootstrap and BLOSUM 80. The Venn diagram was generated using Venny 2.0.2 [77].

### Comparison with existing *Squalus acanthias* resources

*S*. *acanthias* ESTs, nucleotides and protein sequences were downloaded from NCBI on June 29, 2016. Regarding the EST dataset for spiny dogfish 5,824 sequences correspond to an embryo-derived cell line sample [36] (accession SAMN00176998), 15,078 from a study that generated ESTs from a pool of multiple tissues (accession SAMN00175664) and 11,660 correspond to the ESTs generated in two independent studies from the rectal gland (accessions SAMN00150616 and SAMN00154362). In addition, 705 ESTs previously generated in [39] were also downloaded and merged with the ESTs mentioned above and the *S*. *acanthias* nucleotide sequences from NCBI. A non-redundant set of the proteins identified was produced using cd-hit at 90% identity which was then used as query. Tblastn and blastp were run with an e-value cutoff of 1e-06 and 90% identity and the rest of the parameters as default.

### Generation of multiple sequence alignments, phylogenetic trees and mitochondrial signal prediction

Multi-sequence alignments were performed with MUSCLE v3.8.31 [78] using default parameters and visualized with Jalview 2.10.1 [79]. Mitochondrial signals were predicted with TargetP [80] at http://www.cbs.dtu.dk/services/TargetP/ with a cutoff of 0.75.

Multi-sequence alignments were trimmed with trimAl [81] contained in ETE Toolkit 3.0.0b34 [82] with -automated 1 option and the remaining parameters as default. Trimmed alignments were used as input for RaXML version 8.2.9 [83] using the JTT gamma model under the parameters -m PROTGAMMAJTT -f a -x 12345 -N 1000 -T 60 -p 12345 and the remaining options as default. The resulting best trees were visualized with FigTree version 1.4.2 http://tree.bio.ed.ac.uk/software/figtree/.

## Supporting information

S1 Fig Graphical summary of annotation results in the different assemblies. (TIF)

S1 Table Trinotate annotation report for brain assembly. (ZIP)

S2 Table Trinotate annotation report for kidney assembly. (ZIP)

S3 Table Trinotate annotation report for liver assembly. (ZIP)

S4 Table Trinotate annotation report for ovary assembly. (ZIP)

S5 Table Trinotate annotation report for the combined assembly. (ZIP)

S6 Table Urea synthesis transcripts identified in the different assemblies. (XLSX)

S7 Table Urea transport, aquaporins, Rh-glycoproteins and ion channels transcripts identified in the different assemblies. (XLSX)

S8 Table Orthologous groups involved in osmoregulation. Groups were identified by Inparanoid after redundancy removal between elephant shark and spiny dogfish predicted proteins in the different assemblies.
(XLSX)

S9 Table Comparison of the genes identified against the NCBI *Squalus acanthias* nucleotide and protein databases. (XLSX)

S1 File Spiny dogfish full-length cDNA fasta sequences from brain transcriptome (24738 filtered transcripts). (ZIP)

S2 File Spiny dogfish full-length cDNA fasta sequences from kidney transcriptome (21129 filtered transcripts). (ZIP)

S3 File Spiny dogfish full-length cDNA fasta sequences from liver transcriptome (14650 filtered transcripts). (ZIP)

S4 File Spiny dogfish full-length cDNA fasta sequences from ovary transcriptome (18109 filtered transcripts). (ZIP)

S5 File Spiny dogfish predicted protein sequences involved in urea cycle. (TXT)

S6 File Spiny dogfish predicted protein sequences involved in urea transport. (TXT)

S7 File Spiny dogfish predicted protein sequences for in aquaporins. (TXT)

S8 File Spiny dogfish predicted protein sequences for in Rh-glycoproteins. (TXT)

S9 File Tabulated expression values for spiny dogfish brain transcriptome. (ZIP)

S10 File Tabulated expression values for spiny dogfish kidney transcriptome. (ZIP)

S11 File Tabulated expression values for spiny dogfish liver transcriptome. (ZIP)

S12 File Tabulated expression values for spiny dogfish ovary transcriptome. (ZIP)
