# Supplementary figures and images for: Multi-tissue RNA-seq and transcriptome characterisation of the spiny dogfish shark (Squalus acanthias) provides a molecular tool for biological research and reveals new genes involved in osmoregulation
Source: PLoS One. 2017 Aug 23;12(8):e0182756. doi: 10.1371/journal.pone.0182756 (PMC5568229; doi:10.1371/journal.pone.0182756)

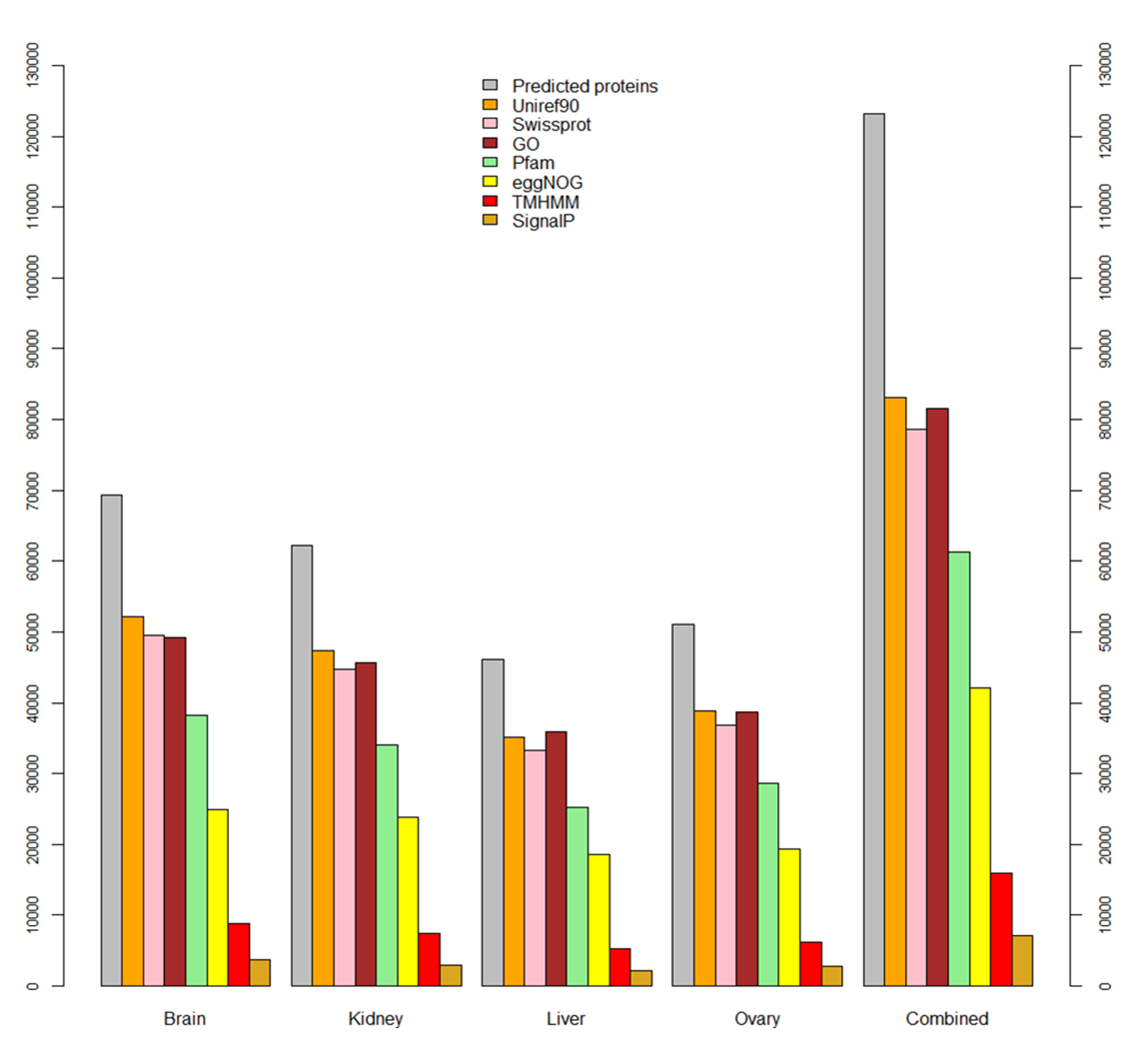

Supplement: S1 Fig — (TIF) [file pone.0182756.s001.tif]
